# Antigen-capture ELISA and immunochromatographic test strip to detect the H9N2 subtype avian influenza virus rapidly based on monoclonal antibodies

**DOI:** 10.1186/s12985-021-01671-4

**Published:** 2021-10-02

**Authors:** Yixin Xiao, Fan Yang, Fumin Liu, Hangping Yao, Nanping Wu, Haibo Wu

**Affiliations:** State Key Laboratory for Diagnosis and Treatment of Infectious Diseases, and National Clinical Research Center for Infectious Diseases, the First Affiliated Hospital, School of Medicine, Zhejiang University, 79 Qingchun Road, Hangzhou, 310003 Zhejiang China

**Keywords:** H9N2 subtype, Avian influenza virus, AC-ELISA, ICT strip, Sensitivity, Specificity

## Abstract

**Background:**

The H9N2 subtype of avian influenza virus (AIV) has become the most widespread subtype of AIV among birds in Asia, which threatens the poultry industry and human health. Therefore, it is important to establish methods for the rapid diagnosis and continuous surveillance of H9N2 subtype AIV.

**Methods:**

In this study, an antigen-capture enzyme-linked immunosorbent assay (AC-ELISA) and a colloidal gold immunochromatographic test (ICT) strip using monoclonal antibodies (MAbs) 3G4 and 2G7 were established to detect H9N2 subtype AIV.

**Results:**

The AC-ELISA method and ICT strip can detect H9N2 subtype AIV quickly, and do not cross-react with other subtype AIVs or other viruses. The detection limit of AC-ELISA was a hemagglutinin (HA) titer of 4 for H9N2 subtype AIV per 100 μl sample, and the limit of detection of the HA protein of AIV H9N2 was 31.5 ng/ml. The ICT strip detection limit was an HA titer of 4 for H9N2 subtype AIV per 100 μl sample. Moreover, both detection methods exhibited good reproducibility and repeatability, with coefficients of variation < 5%. For detection in 200 actual poultry samples, the sensitivities and specificities of AC-ELISA were determined as 93.2% and 98.1%, respectively. The sensitivities and specificities of the ICT strips were determined as 90.9% and 97.4%, respectively.

**Conclusions:**

The developed AC-ELISA and ICT strips displayed high specificity, sensitivity, and stability, making them suitable for rapid diagnosis and field investigation of H9N2 subtype AIV.

## Introduction

Influenza A viruses (Orthomyxoviridae family**)** can infect humans and many other hosts including birds, seals, pigs, cats, horses, and dogs [1, 2]. According to the antigenic characteristics of the hemagglutinin (HA) and neuraminidase (NA**)** surface glycoproteins, influenza A viruses can be further divided into 18 HA and 11 NA subtypes [3]. Avian influenza viruses (AIVs) are influenza A viruses, which usually circulate in wild aquatic birds [4]. AIV causes a wide range of harm to the host, such that highly pathogenic AIV (HPAIV; H5 and H7) infections of poultry usually result in very high mortality. Low pathogenic AIV (LPAIV) infections can induce mild respiratory symptoms and lead to a reduction in poultry production [5]. Studies have shown that the H9N2 subtype of LPAIV is prone to genetic recombination and exchange gene segments with other AIVs, such as H5N1 and H7N9 subtype HPAIVs [6, 7]. Currently, H9N2 AIV has become the most common AIV in Asia [8].

Thus, early and rapid detection and prevention of infection with H9N2 AIV is very important. In 1966, the earliest infection with H9N2 AIV was found in domestic poultry in the USA, and since then, outbreaks of H9N2 AIV have occurred worldwide [5, 9]. In China, the earliest H9N2 AIV infection was found in domestic poultry in Guangdong in 1994 [10]. Subsequently a large-scale outbreak occurred in Hebei province in 1998 and spread to most poultry farms across the country [11]. In the poultry industry, infection with H9N2 AIV caused significant economic losses [12]. Moreover, the H9N2 AIV infection rate remains high in poultry, especially in live poultry markets [13]. Studies have demonstrated that the prevalence of H9N2 AIV in poultry markets and farms in Southeast Asian countries is between 3.5 and 25% [14–19]. Around China, the prevalence of H9N2 AIV is mostly above 10% [20, 21].

Research evidence shows that H9N2 AIV not only infects poultry, but also can infect mammals, including humans [22]. H9N2 AIV infection of humans has been found in certain countries, especially China and Egypt [23, 24]. Previous studies have indicated the H9N2 AIVs have multiple genotypes and their low pathogenicity in poultry makes these viruses easier to spread [24, 25]. At the same time, these viruses have been widely present in mammals (such as Chinese pigs). There have been studies showing that the H9N2 AIV can acquire mutations that enhance receptor binding, toxicity, or transmission ability during the replication of mammalian hosts [22, 26]. Accordingly, some scholars suggested that H9N2 AIV should be considered a potential candidate virus strain for the next pandemic [25, 27]. Thus, the rapid and precise detection of H9N2 AIV is essential.

There are some laboratory methods that can detect H9N2 AIV, such as virus isolation and quantitative real-time reverse transcription polymerase chain reaction (qRT-PCR) [28]. However, virus isolation is time-consuming and entails relatively high environmental requirements. The qRT-PCR method has extremely high sensitivity, but requires special equipment and skilled operators [29]. With the development of molecular biology technology, it has become possible to develop relatively convenient and economical detection methods based on monoclonal antibodies (MAbs). The antigen-capture enzyme linked immunosorbent assay (AC-ELISA) and colloidal gold immunochromatographic test (ICT) strip approaches have been established using MAbs [30, 31]. Many viruses can be detected using MAb detection methods, such as rabies virus, human papillomavirus, and human enterovirus [32–34]. In the field of influenza virus detection, AC-ELISA can be developed to evaluate vaccine efficacy and to diagnose infections, and ICT strips have also been used to detect the AIVs, showing good specificity and sensitivity for samples from patients and poultry infected with AIVs [35–37].

The present study aimed to establish AC-ELISA and ICT strip methods using two MAbs to detect H9N2 AIV.

## Materials and methods

### Viruses and cells

All the viruses used in this study are described in Table 1. All the viruses were obtained from the virus repository in our laboratory [20, 38–41]. AIVs were stored at − 80 °C and propagated at 37 °C using 10-day old chicken embryos, as described previously [20]. Influenza B virus, avian paramyxovirus 4 (APMV-4), infectious bursal disease virus (IBDV), Newcastle disease virus (NDV), and infectious bronchitis virus (IBV) were also acquired from our laboratory virus repository. All viruses were determined using hemagglutinin (HA) and tissue culture infectious dose 50 (TCID50) assays according to standard methods [42]. The experiments involving H5 and H7 subtype AIVs were conducted in an accredited Biosafety Level 3 (BSL-3) containment laboratory at the First Affiliated Hospital of Zhejiang University.

**Table 1:** The specificity and sensitivity of antigen-capture enzyme-linked immunosorbent assay (AC-ELISA) and immunochromatographic test (ICT) strip methods against different viruses

Virus	Subtype	HA	AC-ELISA		Strip	
		Titer	OD value	Test limitation (HA titer)	Result	Test limitation (HA titer)
A/duck /Zhejiang/D4/2018	H9N2	2^6^	1.095	2^2^	+^a^	2^2^
A/chicken/Zhejiang/1026138/2016	H9N2	2^5^	0.986	2^2^	+	2^3^
A/chicken/Zhejiang/13163/2016	H9N2	2^6^	1.254	2^3^	+	2^3^
A/chicken/Zhejiang/221/2016	H9N2	2^7^	1.311	2^2^	+	2^2^
A/chicken/Zhejiang/C1/2013	H9N2	2^7^	1.526	2^2^	+	2^2^
A/pigeon/Zhejiang/2P4/2013	H9N2	2^5^	1.321	2^3^	+	2^2^
A/chicken/Zhejiang/4C91/2013	H9N2	2^5^	1.452	2^3^	+	2^2^
A/quail/Zhejiang/D485/2013	H9N2	2^7^	1.421	2^2^	+	2^2^
A/chicken/Zhejiang/C7195/2013	H9N2	2^5^	1.321	2^2^	+	2^2^
A/chicken/Zhejiang/C497/2013	H9N2	2^7^	1.256	2^2^	+	2^2^
A/chicken/Zhejiang/C55/2013	H9N2	2^7^	1.236	2^2^	+	2^2^
A/chicken/Zhejiang/329/2011	H9N2	2^7^	1.526	2^2^	+	2^2^
A/pigeon/Zhejiang/727044/2014	H9N2	2^5^	1.121	2^3^	+	2^3^
A/pigeon/Zhejiang/2P5/2013	H9N2	2^6^	1.336	2^2^	+	2^3^
A/quail/Zhejiang/A1/2013	H9N2	2^5^	1.201	2^2^	+	2^2^
A/quail/Zhejiang/A2/2013	H9N2	2^6^	1.233	2^3^	+	2^3^
A/quail/Zhejiang/2A6/2013	H9N2	2^5^	1.115	2^2^	+	2^2^
A/egret/Zhejiang/12/2013	H9N2	2^6^	1.334	2^2^	+	2^2^
A/wild duck/Zhejiang/WD5/2014	H9N2	2^6^	1.256	2^2^	+	2^3^
A/duck/Zhejiang/D1/2013	H1N2	2^6^	0.055	−^b^	−	−
A/chicken/Zhejiang/2CP25/2014	H1N3	2^5^	0.046	−	−	−
A/duck/Zhejiang/473/2013	H1N4	2^6^	0.078	−	−	−
A/duck/Zhejiang/6D10/2013	H2N8	2^4^	0.069	−	−	−
A/duck/Zhejiang/4613/2013	H3N2	2^6^	0.065	−	−	−
A/duck/Zhejiang/5/2011	H3N3	2^7^	0.028	−	−	−
A/duck/Zhejiang/D1–3/2013	H3N6	2^6^	0.047	−	−	−
A/duck/Zhejiang/727145/2014	H4N2	2^4^	0.054	−	−	−
A/duck/Zhejiang/409/2013	H4N6	2^5^	0.054	−	−	−
A/goose/Zhejiang/97/2014	H5N1	2^6^	0.039	−	−	−
A/duck/Zhejiang/6DK19/2013	H5N2	2^7^	0.033	−	−	−
A/duck/Zhejiang/6D2/2013	H5N6	2^7^	0.024	−	−	−
A/duck/Zhejiang/W24/2013	H5N8	2^7^	0.027	−	−	−
A/chicken/Zhejiang/1664/2017	H6N1	2^6^	0.065	−	−	−
A/duck/Zhejiang/727038/2014	H6N2	2^4^	0.055	−	−	−
A/chicken/Zhejiang/727018/2014	H6N6	2^5^	0.032	−	−	−
A/duck/Zhejiang/DK16/2013	H7N3	2^5^	0.034	−	−	−
A/chicken/Jiangxi/C25/2014	H7N7	2^7^	0.062	−	−	−
A/chicken/Zhejiang/ ZJU01/2013	H7N9	2^7^	0.035	−	−	−
A/duck/Zhejiang/6D20/2013	H10N2	2^5^	0.041	−	−	−
A/chicken/Zhejiang/8615/2016	H10N3	2^6^	0.077	−	−	−
A/chicken/Zhejiang/2CP2/2014	H10N7	2^6^	0.053	−	−	−
A/chicken/Zhejiang/102622/2016	H10N8	2^5^	0.026	−	−	−
A/duck/Zhejiang/727D2/2013	H11N3	2^3^	0.045	−	−	−
A/duck/Zhejiang/71750/2013	H11N7	2^3^	0.035	−	−	−
Infectious bursal disease virus (IBDV)	NF8	2^5^	0.067	−	−	−
Infectious bronchitis virus (IBV)	H120	2^5^	0.044	−	−	−
Newcastle disease virus (NDV)	La Sota	2^5^	0.032	−	−	−
Avian paramyxovirus 4(APMV-4)	ZJ-1	2^5^	0.058	−	−	−
B/Massachusetts/2/2012	Yamagata	2^5^	0.049	−	−	−

a “+”, positive result

b “−”, negative result

Madin-Darby canine kidney (MDCK) cells and SP2/0 mouse myeloma cells were maintained in our laboratory. Purified HA protein from the H9N2 (A/chicken/Zhejiang/329/2011) subtype AIV was purchased from Sino Biological (Beijing, China) [43].

### Generation and purification of MAbs

BALB/c mice (9 weeks old) were immunized with the purified H9N2 subtype AIV HA protein mixed Freund’s adjuvant (Sigma, St. Louis, MO, USA) intramuscularly, twice at 3 weeks apart. After 6 weeks, the mice were immunized once more with HA protein by tail vein injection. After 3 days, the spleen lymphocytes of the selected mice were fused with SP2/0 cells [43, 44]. The hybridoma cells were screened using a purified H9N2 HA protein-coated ELISA method. The positive monoclonal hybridoma cell line that was obtained after three consecutive limiting dilutions was continuously subcultured and then injected into mice intraperitoneally. To obtain MAbs, a Protein G column (GE Healthcare, Chicago, IL, USA) was used to purify ascites collected from the mice injected with the hybridoma cells [45].

### Isotype and affinity of MAbs

Isotyping of the MAbs was performed using a Monoclonal Antibody Isotyping Kit (Bio-Rad, Hercules, CA, USA). The affinities of each MAb were measured using ELISA, as described previously [45]. In brief, the ELISA plate was coated with purified H9N2 HA protein (20 ng/well) overnight at 4 °C. MAbs were twofold serially diluted, starting at 1 mg/ml, and added to the plate. Incubation was carried out for 1 h at 37 °C. Then, goat anti-mouse IgG (Novus, St Charles, MO USA) was diluted 10,000 times and added as the secondary antibody. Incubation was carried out for 30 min at 37 °C. The color reaction was performed using the 3, 3′, 5, 5′-tetramethylbenzidine (TMB) reagent (KPL, Gaithersburg, MD, USA). After 10 min, the color reaction was stopped using the terminating reagent (KPL). Between each step, phosphate-buffered saline (PBS) with Tween 20 (PBST) was used to wash the plate five times. An ELISA plate reader (Bio-Rad) was read used to read the optical density (OD) at 450 nm, and the antibody’s affinity was estimated as the minimum concentration of the MAb required to provide a positive reaction. The variable genes of the heavy or light chains of the MAbs were sequenced by Sino Biological.

### Immunofluorescence analysis

An immunofluorescence assay (IFA) was used to visualize the binding of the MAbs to the virus-infected MDCK cells [46, 47]. After incubation with the virus for 24 h, virus-infected MDCK cells were fixed with paraformaldehyde. Thereafter, the MDCK cells were permeabilized using Triton X-100. Then, MAbs 3G4 or 2G7 were added and incubated for 1 h at 37 °C. The goat anti-mouse IgG heavy plus light chain (H + L)-Alexa Fluor (Abcam, Cambridge, UK) was then added. The wells were washed with PBS three times between each step. The results were scored using an EVOS M7000 instrument (Thermo Fisher Scientific, Waltham, MA, USA).

### Preparation of H9N2 AC-ELISA

**Fig. 1:**
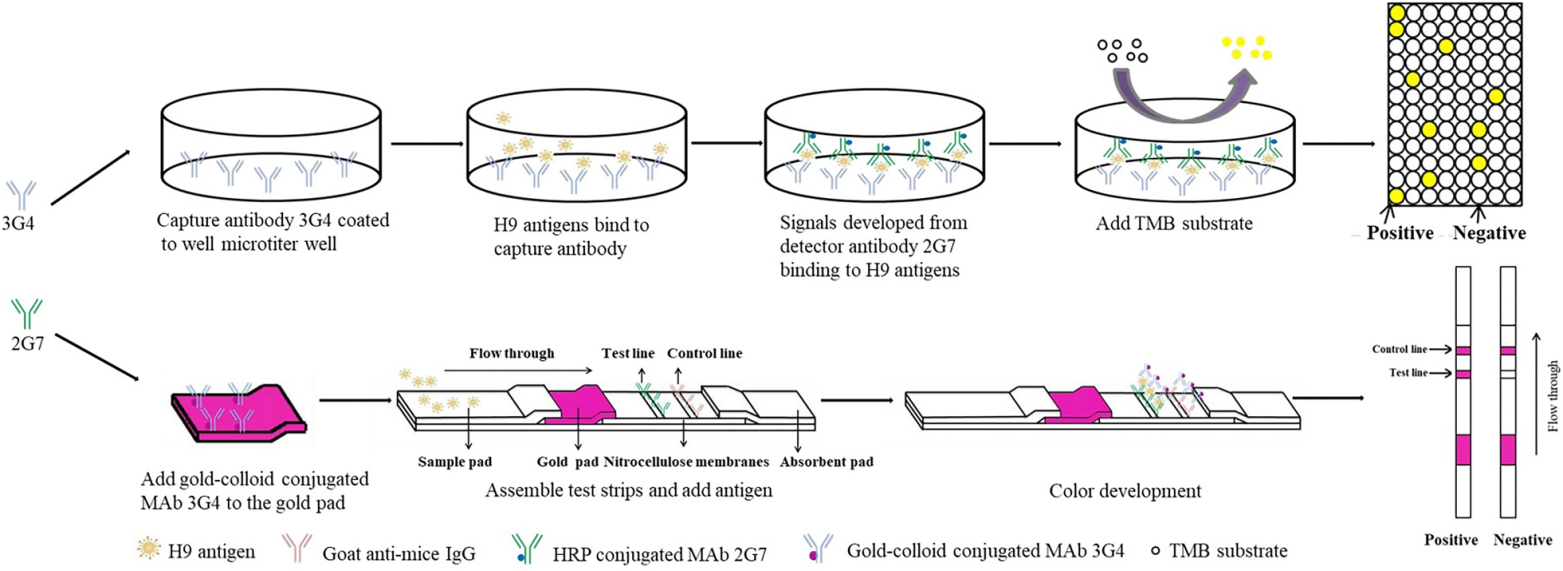
Procedures for the AC-ELISA and ICT strips

The procedure for AC-ELISA (Fig. 1) was described previously [35, 48]. In brief, based on the results of MAb affinity measurements, MAb 3G4 was selected for capture and used to coat a 96-well ELISA plate at 80 ng/well in 100 μl of coating buffer at 4 °C. After 12 h, MAb 2G7, which was selected as the detection antibody, was labelled with horseradish peroxidase (HRP; Innoreagents, Huzhou, China). Then, the ELISA plate was washed and blocked with bovine serum albumin (BSA). After washing, samples were added into the ELISA plate and incubated for 1 h at 37 °C. Then, after washing the plate, 2G7-MAb-HRP (4 μg/ml) was added and incubated for 30 min at 37 °C. The plate was then washed and TMB solution was added at 100 μl/well. After 10 min, the TMB stop solution was added. The OD value (450 nm) was then detected using an ELISA reader. An OD value greater than 2.1 times that of the negative control was considered to indicate a positive reaction.

### ICT strip preparation

The procedure for using the ICT strip is shown in Fig. 1. The preparation of the colloidal gold solution was described previously [37, 49]. In brief, 0.01% HAuCl4 solution was heated to 100 °C, and then a trisodium citrate solution was added quickly with continuous vigorous stirring. Then, the colloidal gold solution was continuously boiled until the color changed to wine-red. After cooling, the pH of the solution was adjusted to 7.2 using potassium carbonate. Then, 10 ml of the colloidal gold solution placed into a glass bottle into which 100 μl MAb 3G4 (1 mg/ml) was added. Incubation was carried out for 30 min with gentle stirring. After blocking with BSA, the solution was centrifuged for 30 min at 4 °C. The colorless supernatant was discarded and the pellet was re-dissolved with 1 ml PBST (containing 1% BSA).

The ICT strip contained an absorbent pad, nitrocellulose (NC) membranes, a MAb-gold conjugated, pad and a sample pad. The NC membranes were coated with MAb 2G7 as the test line and with goat anti-mouse IgG (Solarbio, Beijing, China) as the control line.

### Sensitivity, specificity, and repeatability of the H9N2 AC-ELISA and ICT strips

The specificity of the assays was tested using different subtypes of AIV (19 H9N2 AIVs and 25 other AIV subtypes), and other viruses (IBDV, IBV, APMV-4, NDV, and influenza B virus). To determine the sensitivity, twofold serial dilutions of H9N2 AIV allantoic fluid and purified H9N2 proteins were used. A twofold serial dilution of H10N7 AIV allantoic fluid was used as the negative control. To evaluate repeatability, all samples were tested in triplicate, and all assays were repeated three times.

### Assessment of the ability to rapidly detect actual poultry samples

To assess the clinical application of the two methods, 200 cloacal swabs (100 from chickens and 100 from ducks) collected from 10 poultry farms in Zhejiang Province were detected using multiplex qRT-PCR, AC-ELISA, and ICT strip methods. There were 20 samples for every farm. An Influenza A virus universal PCR kit (Liferiver Bio-Tech, Shanghai, China) was used to perform the qRT-PCR assay [37].

## Results

### Isotype and affinity of the MAbs

Five murine MAbs (3G4, 2G7, 1B12, 1C12, and 2F1) were screened using ELISA. MAbs 3G4, 2G7, 1B12, and 1C12 belonged to the IgG1 subclass, and Mab 2F1 belonged to the IgG2a subclass. Among these MAbs, MAbs 3G4 and 2G7 were chosen to develop the detection method because of their high affinity (Table 2). Although none of the five MAbs displayed hemagglutination inhibition (HI) or virus neutralization (VN) activity against H9N2 subtype AIV, these MAbs reacted with all H9N2 subtype AIVs available in our laboratory (Table 1). Therefore, MAbs 3G4 and 2G7 were complementary to each other and hence likely to be suitable for rapid detection of H9N2 subtype AIVs.

**Table 2:** The characteristics of two monoclonal antibodies (MAbs) used to establish the detection methods

MAbs	Isotype^a^		Affinity (μg/ml)	CDR3^b^	
	subclass	Type		Heavy chain	Light chain
2G7	IgG1	Κ	15.63	ARLTGTDY	QQGDFIPRT
3G4	IgG1	Κ	15.63	ESQRG	WQGTHFPYT

^a^ The immunoglobulin isotypes of MAbs

^b^ Complementarity-determining region

### Immunofluorescence analysis

**Fig. 2:**
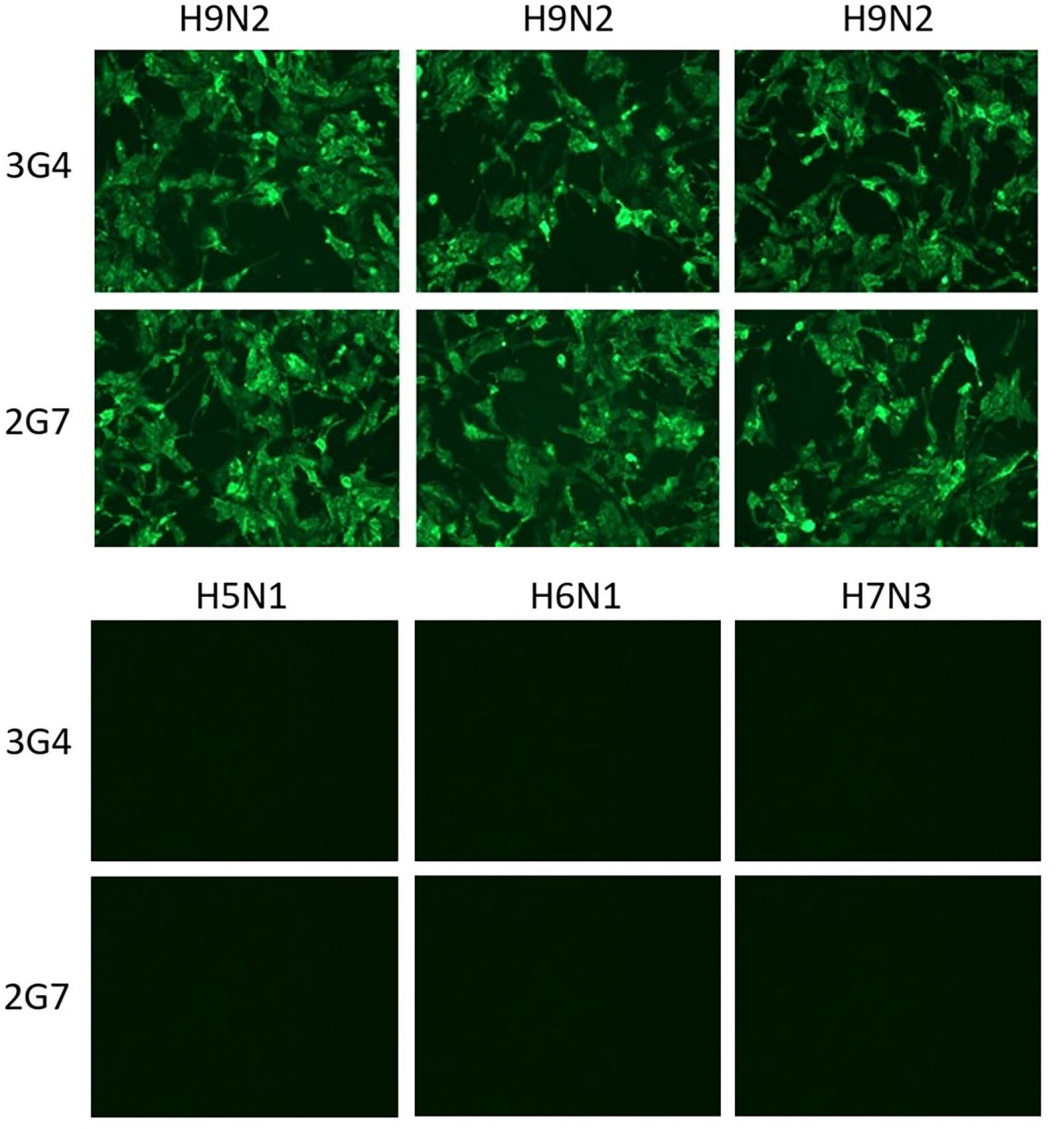
IFA results for MAbs 3G4 and 2G7. Neither MAb exhibited non-specific binding to MDCK cells infected with H5N1, H6N1, and H7N3 subtype AIVs. The two MAbs showed strong reactivity toward MDCK cells infected with the H9N2 subtype AIV

IFA was used to analyze whether the MAbs could recognize H9N2 subtype AIV in MDCK cells. Neither MAb exhibited non-specific binding to MDCK cells infected with H5N1, H6N1, and H7N3 subtype AIVs; However, MAbs 3G4 and 2G7 showed strong reactivity toward MDCK cells infected with H9N2 subtype AIV (Fig. 2).

### Assessment of the AC-ELISA

**Fig. 3:**
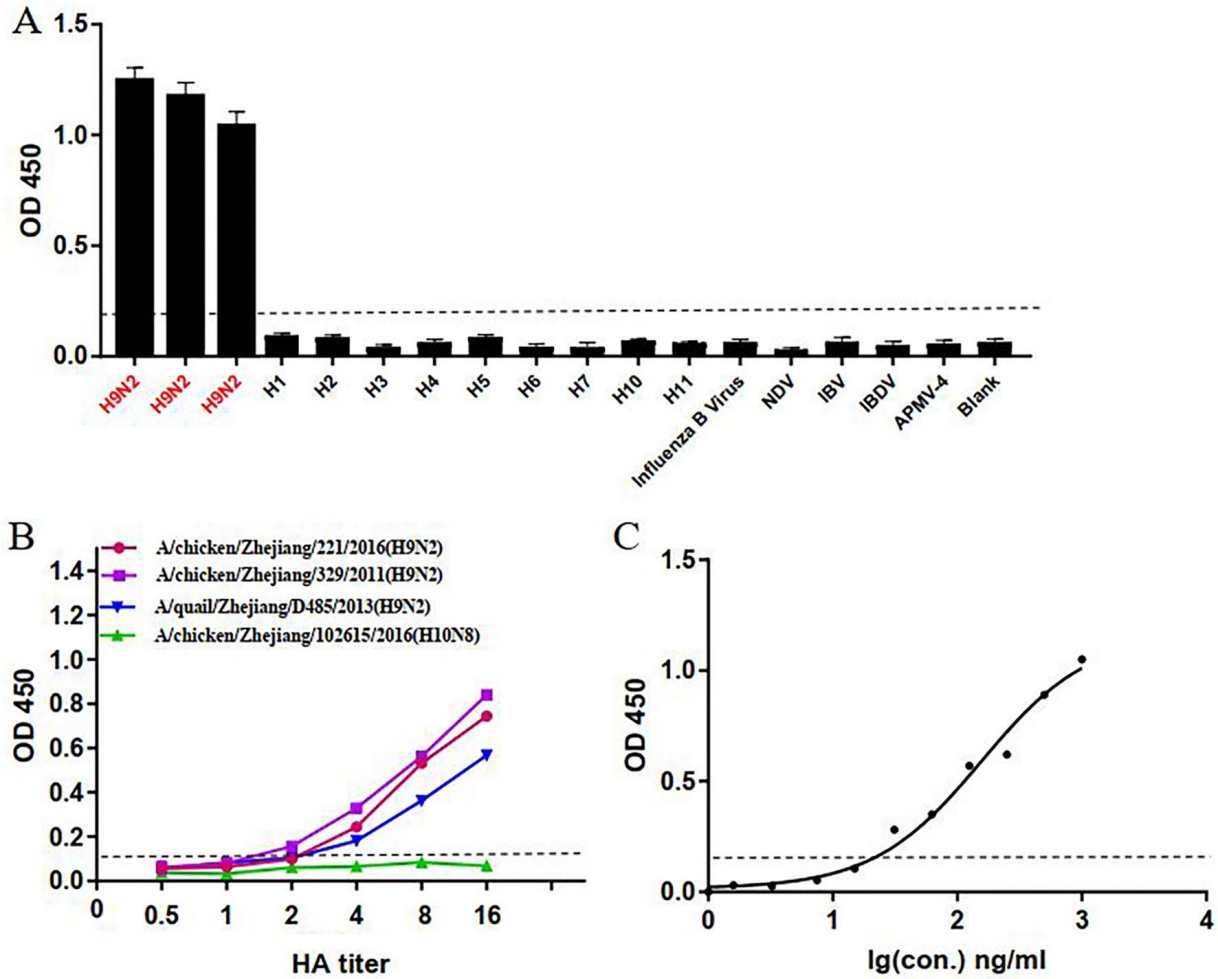
Assessment of AC-ELISA. **a** To determine the specificity, samples of 100 μl of the H9N2 subtype AIVs or non-H9N2 viruses (OD value) allantoic fluid were measured and averaged from three independent tests. **b** To test the sensitivity, three different H9N2 subtype AIVs and one H10N8 subtype AIV (negative control) at an HA titer of 2^4^ were twofold serially diluted. **c** Standard curve of AC-ELISA for the purified H9 HA protein (A/chicken/Zhejiang/329/2011). The limit of detection was 31.5 ng/ml. The OD was measured at 450 nm, and the cut-off value is denoted by a dotted line

To determine its specificity, AC-ELISA was tested using different strains of viruses, including H9N2 subtype AIVs and other virus strains (Table 1 and Fig. 3a). No cross-reactivity was observed for any of the other subtypes of influenza A virus (H1, H2, H3, H4, H5, H6, H7, H10, and H11) or for the other viruses tested (NDV, APMV-4, IBV, IBDV, and influenza B virus).

To determine the sensitivity of AC-ELISA, three different H9N2 subtype AIVs were assessed alongside the H10N8 subtype AIV as a negative control. The four selected viruses were twofold serially diluted to determine the detection limit (Table 1 and Fig. 3b). The detection limits were 4 HA titer in 100 μl of virus sample (A/chicken/Zhejiang/329/2011, A/quail/Zhejiang/D485/2011 and A/chicken/Zhejiang/221/2011). The detection limit of AC-ELISA for the H9N2 HA protein was 31.5 ng/ml (Fig. 3c).

To evaluate repeatability, twofold serially diluted H9N2 subtype AIV was detected (A/chicken/Zhejiang/329/2011). In the intra- and inter-batch repeatability tests, the coefficient of variation (CV%) was < 5% (Table 3 and Table 4), which showed that the AC-ELISA method possessed good reproducibility.

**Table 3:** Intra-batch variation in antigen-capture enzyme-linked immunosorbent assay (AC-ELISA) detection of the H9N2 subtype avian influenza virus

HA titer	OD value in intra-batch			Mean ± SD	CV%^a^
	1	2	3		
2^5^	1.105	1.132	1.112	1.116 ± 0.014	1.3
2^4^	0.825	0.853	0.844	0.841 ± 0.014	1.7
2^3^	0.552	0.564	0.572	0.563 ± 0.010	1.8
2^2^	0.322	0.342	0.325	0.330 ± 0.011	3.3
2^1^	0.153	0.163	0.156	0.157 ± 0.005	2.9
2^0^	0.082	0.078	0.082	0.081 ± 0.002	2.9

^a^CV%, coefficient of variation

**Table 4:** Inter-batch variation in antigen-capture enzyme-linked immunosorbent assay (AC-ELISA) detection of the H9N2 subtype avian influenza virus

HA titer	OD value in inter-batch			Mean ± SD	CV%^a^
	1 day	30 days	60 days		
2^5^	1.197	1.116	1.138	1.150 ± 0.042	3.6
2^4^	0.841	0.861	0.872	0.858 ± 0.016	1.9
2^3^	0.563	0.565	0.535	0.554 ± 0.017	3.0
2^2^	0.330	0.337	0.358	0.341 ± 0.015	4.3
2^1^	0.157	0.155	0.168	0.160 ± 0.007	4.3
2^0^	0.081	0.082	0.077	0.080 ± 0.003	3.4

^a^CV%, coefficient of variation

### Assessment of the ICT strip assay

**Fig. 4:**
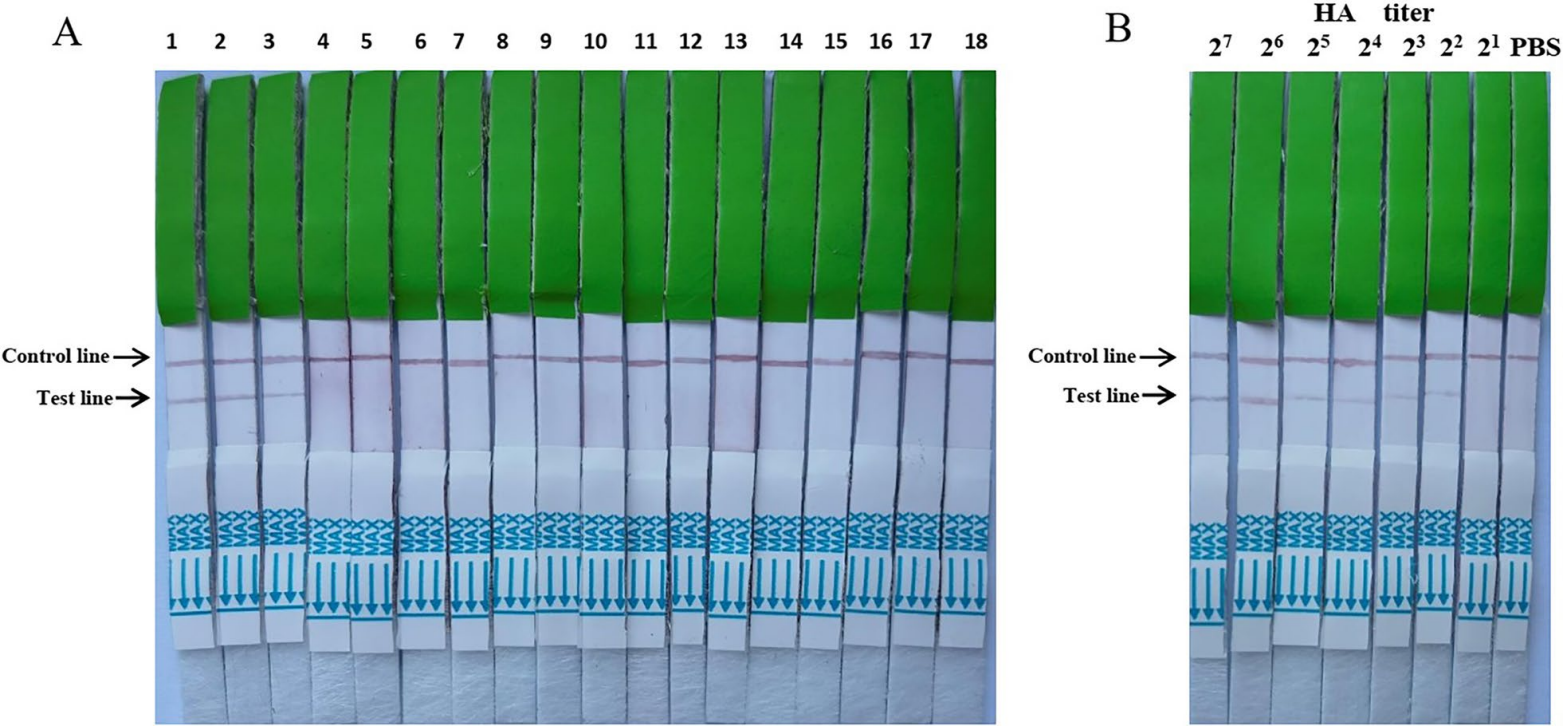
Assessment of the ICT strip. **a** Specificity of the ICT strip method. 1–3 = H9N2; 4–12 = H1, H2, H3, H4, H5, H6, H7, H10, and H11 subtype AIVs; 13–17 = Influenza B virus, NDV, IBV, IBDV, and APMV-4; 18 = negative control (PBS). **b** Sensitivity of the ICT strip method tested using serial twofold diluted allantoic fluid of the H9N2 subtype AIV (A/chicken/Zhejiang/329/2011) ranging from an HA titer of 2^7^ to 2^1^

The specificity of the ICT strip was tested using H9N2 viruses and other non-H9N2 viruses, as described above. Only the H9N2 subtype AIV samples showed positive results (Fig. 4a). The result suggested that the ICT strip could specifically detect H9N2 subtype AIVs (Table 1).

Two-fold serially diluted allantoic fluid from H9N2 subtype AIV (A/chicken/Zhejiang/329/2011)-infected chicken eggs was used to test the sensitivity of the strip. The detection limit of the ICT strip was 100 μl of allantoic fluid containing a 4 HA titer of the virus or a median tissue culture infectious dose (TCID50) of 10^3.15^ (Fig. 4b).

To verify their stability, the ICT strips were tested after being assembled for 30 and 60 days, and the results revealed comparable specificity and sensitivity to newly assembled ICT strips.

### Actual application of AC-ELISA and ICT strip methods

To assess the actual clinical application of the methods, cloacal swabs were collected from poultry in farms and subjected to analysis by qRT-PCR, AC-ELISA, and ICT strips (Table 5). We used the results of qRT-PCR as the standard, using which, 44 samples were determined to be positive. By comparison, the results of AC-ELISA showed that 41 of the 200 poultry samples were positive, and the ICT strip assay result identified 40 of 200 poultry samples as positive. Furthermore, the positive samples identified by qRT-PCR and AC-ELISA were confirmed to be the same samples. The sensitivity and specificity of the AC-ELISA method were calculated as 93.2% (41/44) and 98.1% (153/156), respectively. The sensitivity and specificity of the ICT strips were 90.9% (40/44) and 97.4% (152/156), respectively. These results indicated that both AC-ELISA and ICT strip methods exhibited high sensitivity and specificity for environmental sample detection.

**Table 5:** The result of detecting poultry samples using AC-ELISA, ICT strip and qRT-PCR methods

Number of samples	AC-ELISA		Strip	
	Positive	Negative	Positive	Negative
*qRT-PCR*				
Positive (44)	41	3	40	4
Negative (156)	3	153	4	152
Sensitivity	93.2% (41/44)		90.9% (40/44)	
Specificity	98.1% (153/156)		97.4% (152/156)	

## Discussion

Epidemic of H9N2 subtype AIVs have caused direct financial losses to the poultry industry and threatened human health [50]. Since the 1990s, many countries have gradually begun to immunize poultry with specific vaccines [51]. However, epidemiological studies have shown that H9N2 AIV is still ubiquitous in poultry around the world and has become an endemic disease [9]. Furthermore, although infections with the H9N2 AIV only induce mild respiratory symptoms, its causes large losses to the poultry industry [50]. Therefore, the establishment of rapid detection methods for H9N2 AIV will be of great significance in monitoring its infection and spread.

The AC-ELISA and ICT strip methods were developed to rapidly detect the H9N2 AIVs based on suitable MAbs. MAbs 3G4 and 2G7 were selected because of their high affinity. Although MAbs 3G4 and 2G7 did not show HI and VN activities, they reacted specifically with H9 HA antigens. This indicated that the MAbs might bind to a linear epitope on the HA antigen, which meets the diagnostic requirements [44]. Therefore, the MAbs were used to establish the H9N2 subtype detection method employed in the AC-ELISA and ICT strip methods.

An ICT strip to detect one strain of H9N2 virus was developed previously and showed good sensitivity and specificity [44]. However, an AC-ELISA method to detect H9N2 AIV has not been reported. Thus, the developed AC-ELISA method could provide a new option to detect H9N2 AIV rapidly. In this study, the AC-ELISA and ICT strip methods showed good sensitivity, specificity, and repeatability. The detection sensitivity of AC-ELISA and ICT strips were 4 HA titer for 100 μl samples, and neither showed cross-reactivity with any of the non-H9N2 viruses.

In the detection of actual poultry samples, the AC-ELISA method achieved slightly higher specificity and sensitivity than the ICT strips [45]. This might be related to the visual identification method used in the ICT strip. However, the ICT strip is more convenient to carry and the results can be displayed within 10 min. Thus it is more suitable for field investigation [52].

## Conclusion

In conclusion, the AC-ELISA and ICT strip methods were established using two MAbs to detect H9N2 subtype AIVs rapidly. The two methods have good specificity and sensitivity, and possessing important application value for the rapid detection of viral diseases, and could be further applied in clinical practice.

## Data Availability

The dataset used and analyzed during the current study is available from the corresponding author upon reasonable request.

## Abbreviations

AIV: Avian influenza virus
AC-ELISA: Antigen-capture enzyme-linked immunosorbent assay
ICT: Immunochromatographic test
HA: Hemagglutinin
NA: Neuraminidase
qRT-PCR: Quantitative real-time reverse transcription-polymerase chain reaction
MAb: Monoclonal antibody
NDV: Newcastle disease virus
APMV-4: Avian paramyxovirus-4
IBV: Infectious bronchitis virus
IBDV: Infectious bursal disease virus
MDCK: Madin-Darby canine kidney
BSA: Bovine serum albumin
TMB: Tetramethylbenzidine
HRP: Horseradish peroxidase
PBS: Phosphate buffered saline
PBST: Phosphate buffered saline–0.1% Tween 20
CV: Coefficient of variation
